# Regulation of the inflammatory profile of stromal cells in human breast cancer: prominent roles for TNF-α and the NF-κB pathway

**DOI:** 10.1186/s13287-015-0080-7

**Published:** 2015-05-01

**Authors:** Christina Katanov, Shalom Lerrer, Yulia Liubomirski, Leonor Leider-Trejo, Tsipi Meshel, Jair Bar, Rotem Feniger-Barish, Iris Kamer, Gali Soria-Artzi, Hadar Kahani, Debabrata Banerjee, Adit Ben-Baruch

**Affiliations:** Department of Cell Research and Immunology, George S. Wise Faculty of Life Sciences, Tel Aviv University, P.O. Box 39040, Tel Aviv, 6997801 Israel; Department of Pathology, Tel Aviv Sourasky Medical Center and the Sackler School of Medicine, Tel Aviv University, 6 Weizmann Street, Tel Aviv, 64239 Israel; Institute of Oncology, Sheba Medical Center, Tel-Hashomer, Ramat Gan, 5262100 Israel; Department of Medicine and Pharmacology, Robert Wood Johnson Medical School and Graduate School of Biomedical Sciences, Rutgers, The State University of New Jersey, 195 Little Albany Street, New Brunswick, NJ 08901 USA

## Abstract

**Introduction:**

Breast cancer progression is promoted by stromal cells that populate the tumors, including cancer-associated fibroblasts (CAFs) and mesenchymal stem/stromal cells (MSCs). The activities of CAFs and MSCs in breast cancer are integrated within an intimate inflammatory tumor microenvironment (TME) that includes high levels of tumor necrosis factor α (TNF-α) and interleukin 1β (IL-1β). Here, we identified the impact of TNF-α and IL-1β on the inflammatory phenotype of CAFs and MSCs by determining the expression of inflammatory chemokines that are well-characterized as pro-tumorigenic in breast cancer: CCL2 (MCP-1), CXCL8 (IL-8) and CCL5 (RANTES).

**Methods:**

Chemokine expression was determined in breast cancer patient-derived CAFs by ELISA and in patient biopsies by immunohistochemistry. Chemokine levels were determined by ELISA in (1) human bone marrow-derived MSCs stimulated by tumor conditioned media (Tumor CM) of breast tumor cells (MDA-MB-231 and MCF-7) at the end of MSC-to-CAF-conversion process; (2) Tumor CM-derived CAFs, patient CAFs and MSCs stimulated by TNF-α (and IL-1β). The roles of AP-1 and NF-κB in chemokine secretion were analyzed by Western blotting and by siRNAs to c-Jun and p65, respectively. Migration of monocytic cells was determined in modified Boyden chambers.

**Results:**

TNF-α (and IL-1β) induced the release of CCL2, CXCL8 and CCL5 by MSCs and CAFs generated by prolonged stimulation of MSCs with Tumor CM of MDA-MB-231 and MCF-7 cells. Patient-derived CAFs expressed CCL2 and CXCL8, and secreted CCL5 following TNF-α (and IL-1β) stimulation. CCL2 was expressed in CAFs residing in proximity to breast tumor cells in biopsies of patients diagnosed with invasive ductal carcinoma. CCL2 release by TNF-α-stimulated MSCs was mediated by TNF-RI and TNF-RII, through the NF-κB but not *via* the AP-1 pathway. Exposure of MSCs to TNF-α led to potent CCL2-induced migration of monocytic cells, a process that may yield pro-cancerous myeloid infiltrates in breast tumors.

**Conclusions:**

Our novel results emphasize the important roles of inflammation-stroma interactions in breast cancer, and suggest that NF-κB may be a potential target for inhibition in tumor-adjacent stromal cells, enabling improved tumor control in inflammation-driven malignancies.

**Electronic supplementary material:**

The online version of this article (doi:10.1186/s13287-015-0080-7) contains supplementary material, which is available to authorized users.

## Introduction

The development and progression of breast tumors are multifactorial processes that are influenced by the tumor microenvironment (TME). Recent studies demonstrated that breast tumors are populated by myofibroblasts that express pro-cancerous functions [1-4], known as cancer-associated fibroblasts (CAFs). Various origins of these cells may exist, including resident tissue fibroblasts and mesenchymal stem/stromal cells (MSCs) that have been continuously exposed to tumor-derived and TME constituents. Such MSCs, originating in bone marrow (BM) or adipose tissues generally have pro-cancerous effects that promote malignancy in many tumor systems, including breast cancer [5-12]. *In vitro*, tumor cell products that are present in tumor-derived conditioned medium (CM) convert MSCs to CAFs which become fully and potently functional in promoting malignancy *in vivo* [11-14].

The activities of CAFs and MSCs do not take place in the void, but rather are integrated in their intimate TME. In many cancers, the TME is dominated by inflammatory elements, including inflammatory leukocytes and inflammatory soluble factors that generally promote disease progression [15-18]. The inflammatory cytokines tumor necrosis factor alpha (TNF-α) and interleukin 1β (IL-1β) are often present in the inflammatory milieu of many tumors. In contrast to tumor-cytotoxic effects caused by acute local TNF-α administration, chronic and persistent presence of TNF-α in tumors has strong pro-tumoral effects in many cancers [19-21]. Accordingly, inhibition of TNF-α or its receptors has prominent anti-tumor effects in animal models of breast cancer [22-29]. In parallel, major causative pro-tumoral roles were attributed to IL-1β in breast cancer *via* angiogenesis and matrix-remodeling activities [30-37]. Overall, based on recent studies addressing the roles of TNF-α and IL-1β in malignancy, both cytokines are now considered potential targets for therapy in cancer [32,38-40].

We recently reported that TNF-α and IL-1β were minimally expressed by normal breast epithelial cells, but were highly expressed in tumor cells of biopsies from most breast cancer patients [41]. In such individuals, the elevated expression of TNF-α and IL-1β was significantly correlated with relapse and advanced disease [41-49]. Despite emerging information on the impact of these inflammatory cytokines on tumor-promoting events in stromal cells [10,50-55], their ability to shape the inflammatory phenotype of CAFs and MSCs has been only partly revealed.

Recent studies indicate that CAFs and MSCs promote malignancy through the expression of inflammatory chemokines [4,54-65]. In this respect, inflammatory chemokines such as CCL2 (monocyte chemoattractant protein 1 MCP-1), CXCL8 (IL-8) and CCL5 (RANTES) are of major relevance because they promote aggressiveness in tumor cells, they induce tumor-supporting effects in cells of the TME, and they play direct roles in advancing tumor growth and metastasis in many cancer diseases, including cancer of the breast [21,66-70]. MSC-derived and CAF-derived inflammatory chemokines promote tumor progression by inducing the infiltration of pro-tumorigenic myeloid cells to tumors (such as tumor-associated macrophages (TAMs) and myeloid-derived suppressor cells (MDSCs) [17,67,71-75]), increasing angiogenesis, elevating tumor cell stemness, invasion and proliferation, and promoting the recruitment of MSCs to primary tumors and metastases [4,54-65]. Overall, the outcome of such chemokine activities is a pronounced promotion of cancer progression and tumor cell dissemination to distant organs.

Our overall goal in this study was to investigate aspects of inflammation–stroma interactions in breast cancer. To this end, we determined the influence of breast tumor-derived factors and of inflammatory cytokines on the inflammatory phenotype of CAFs and MSCs, manifested by the release of the pro-cancerous chemokines CCL2, CXCL8 and CCL5 by these cells. The novel findings obtained in this study show intensive inflammation–stroma interactions that act specifically through the designated pathway of nuclear factor κB (NF-κB) activation in stromal cells. Through these interactions, the inflammatory microenvironment shapes the tumor-promoting phenotype of MSCs and CAFs and may thus enhance tumor progression.

## Methods

### Breast tumor cell cultures

Human breast tumor MDA-MB-231 cells were obtained from ATCC (Manassas, VA, USA) and MCF-7 cells were kindly provided by Prof. Kaye, Weizmann Institute of Science, Rehovot, Israel. The MCF-7 cells were authenticated on the basis of published characteristics of MCF-7 cells [76,77] by verifying that they express an active estrogen receptor alpha, respond to estrogen, express low levels of ErbB2, form tumors upon supplementation of estrogen and matrigel, and have low metastatic potential. These cells were grown in enriched Dulbecco’s modified Eagle’s medium (DMEM), including 10% fetal bovine serum (FBS), 100 U/ml penicillin, 100 μg/ml streptomycin, 250 ng/ml amphotericin, and 4 mM l-glutamine (Biological Industries, Beit Ha’emek, Israel).

### Isolation of CAFs from patient breast tumors

CAFs were obtained from surgically resected breast tumors of patients who provided written informed consent as approved by the Institutional Review Board of Sheba Medical Center. Tissues were cut into small pieces, shaken overnight at 37°C in collagenase type 4 (250 U/ml, #S3J6523; Worthington Biochemical, Lakewood, NJ, USA) in DMEM, filtered (100 mm cell strainer; BD Biosciences, San Jose, CA, USA) and plated in DMEM supplemented with 20% FBS, 1 mM sodium pyruvate, 2 mM l-glutamine, minimum essential medium nonessential amino acids, antibiotics and 60 mM β-mercaptoethanol (Biological Industries; and Sigma-Aldrich, St. Louis, MO, USA). Cells propagated in these conditions had typical fibroblastic phenotype: their identity was verified by the absence of the epithelial marker cytokeratin and the presence of the typical mesenchymal markers vimentin, α-smooth muscle actin (αSMA), fibroblast-specific protein 1 (FSP1) and fibroblast-activation protein alpha (FAPα) (Figure S1 in Additional file 1). The cells were routinely grown in medium as above or in Eagle’s minimum essential medium supplemented with 10 to 20% FBS with regular tissue culture additives.

Two human patient-derived CAF isolates were generated and characterized for CAF phenotype as described above. CAFs #1 cells were derived from a lung metastasis of breast cancer and were immortalized by human telomerase reverse transcriptase (hTERT), as described previously [78]. CAFs #2 cells were derived from a primary breast tumor of a different patient and were kept in culture without hTERT immortalization.

### Origins and growth of MSCs

Two sources of human BM-derived MSCs of healthy individuals were used in the study. First, in the earlier stages of the study, human MSCs were isolated from unprocessed BM of healthy individuals obtained from Lonza (Walkersville, MD, USA; Cambrex at that time) and were cultured in mesencult medium (#05411; Stemcell Technologies Inc., Vancouver, BC, Canada). Following establishment in culture, the isolated MSCs were cultured in α-minimum essential medium containing 10% FBS and penicillin-streptomycin (as in [13]). In line with typical characteristics of MSCs, the isolated cells expressed CD44, CD105, CD90, HLA-ABC and Stro1, while they were negative for CD45, HLA-DR and CD11b (see [13]; data not shown), as determined by flow cytometry using FITC-labeled antibodies (BD Biosciences). Expanded cultures of human BM-derived MSCs were analyzed for adipogenic, osteogenic and myogenic differentiation *in vitro*, to determine multipotency (data not shown). Second, at the advanced stages of the study, fully characterized MSCs were purchased from Lonza. As indicated in Lonza’s datasheet, the cells had typical MSC characteristics. They expressed CD44, CD29, CD105, and CD166 and did not express CD45, CD14 and CD34. Also, the cells can differentiate to adipogenic, chondrogenic and osteogenic lineages when cultured in the recommended differentiation medium. The cells were thawed into enriched mesencult medium (Stemcell Technologies Inc.) and then were subcultured every 5 to 7 days in enriched DMEM medium. In all cases, cell growth was continued in DMEM medium for a limited number of passages (generally up to 10 passages).

### Long-term growth of MSCs with Tumor CM, followed by TNF-α stimulation

MDA-MB-231 and MCF-7 breast tumor cells were grown in enriched DMEM culture medium (as above). Tumor CM from these cancer cells was collected after 18 to 24 hours, centrifuged at 1,200 rpm for 5 minutes and supernatants were passed through sterile filters with 0.45 μm polyvinylidene difluoride membrane (Millipore, Bedford, MA, USA). MSCs were exposed to such fresh Tumor CM twice a week for a ~30-day time period, following the protocol described in [13] that has generated CAFs which promoted tumor growth *in vivo* in past experiments [13]. In parallel, control MSCs from the same initiating cell stock were cultured with enriched DMEM medium. To verify the CAF-like phenotype of cells generated by this process (termed herein CAFs), in this study we used criteria of modified morphology and typical CAF markers: the expression of vimentin, upregulation of αSMA expression (Figure S2 in Additional file 1) and elevated expression of FSP1 (performed for MCF-7 CM only; data not shown).

CAFs obtained by stimulating MSCs for ~30 days and their control MSCs were trypsinized, centrifuged and counted. The cells were plated at equivalent density and, following an additional 24 hours of incubation, supernatants from the differentiated CAFs and from control MSCs were frozen in −20°C. In parallel, an aliquot of the original Tumor CM was frozen at −20°C. All supernatants were then thawed at the same time (from CAFs obtained by incubation with Tumor CM, from control MSCs that were grown for the same time period and the CM of the tumor cells themselves) for determination of CCL2, CXCL8 and CCL5 levels in these supernatants by enzyme-linked immunosorbent assay (ELISA).

In other assays, this same procedure was utilized in the presence of short stimulation by TNF-α (50 ng/ml; PeproTech, Rocky Hill, NJ, USA), carried out during the last 24 hours of culture. TNF-α working concentrations were chosen based on criteria described next in Stimulation of MSCs by TNF-α or IL-1β. As control for TNF-α, the cells were incubated with the vehicle of the cytokine (0.1% bovine serum albumin (BSA)). CCL2, CXCL8 and CCL5 levels in cell supernatants were determined by ELISA.

### Stimulation of MSCs by TNF-α or IL-1β

MSCs were cultured for 24 hours in enriched DMEM medium. The medium was then removed, and cells were incubated for 24 hours in medium containing TNF-α (50 ng/ml) or IL-1β (500 pg/ml) (PeproTech). Cytokine working concentrations were chosen based on calibrations performed in other cell systems in our laboratory and are in the accepted range used *in vitro* in other studies (several selected publications [79-81]). Chemokine expression in cell supernatants was determined by ELISA. In controls, we verified that the vehicle of the cytokines (0.1% BSA) did not induce chemokine release by the MSCs.

In specific cases, neutralizing antibodies or small interfering RNAs (siRNAs) were used. For experiments with antibodies to TNF-α receptors, MSCs were plated in enriched DMEM medium for 24 hours. The cells were then washed and incubated in DMEM medium supplemented with 1% FBS, in the presence of neutralizing antibodies for tumor necrosis factor receptors TNF-RI and TNF-RII (10 μg/ml, #MAB225 and #MAB726, respectively; R&D Systems, Minneapolis, MN, USA) or isotype-matched nonrelevant antibody control (10 μg/ml, #400101; BioLegend, Roselle, San Diego, USA) for 1 hour. Antibody concentrations were chosen based on preliminary analyses, in which several concentrations of neutralizing antibodies were tested (data not shown). Following this incubation period, TNF-α (50 ng/ml) or its vehicle (as above) was added to cells. After 24 hours, cell supernatants were collected for ELISA assays.

For experiments with siRNAs to c-Jun or p65, MSCs were plated in enriched DMEM medium for 24 hours. The cells were then transiently transfected with siRNA to c-Jun (60 nM, #L-003268-00; Dharmacon, Lafayette, CO, USA), siRNA to p65 (30 nM, #MU-003533-02; Dharmacon; kindly provided by Prof. Wiemann, DKFZ, Heidelberg Germany) or control siRNA (same concentrations) by Lipofectamine RNAiMAX™ transfection reagent (Life Technologies, Grand Island, NY, USA) according to the manufacturer’s instructions. The cells were washed and incubated in DMEM medium overnight, and then stimulated with TNF-α (25 ng/ml; a suboptimal concentration of TNF-α was used in order to facilitate detection of inhibitory effects) for 24 to 48 hours. Cell supernatants were collected for ELISA assays.

### MSC stimulation by CM derived from MDA-MB-231 cells, transfected with TNF-α-expressing vector

MDA-MB-231 cells were transiently transfected with pcDNA3.1 vector or with the same vector coding for human TNF-α, by ICAFectin™ 441 DNA transfection reagent (InCellArt, Nantes, France) according to the manufacturer’s instructions. After 24 hours, Tumor CM was harvested and TNF-α expression was determined by ELISA, as described below. In parallel, on the day of MDA-MB-231 cell transfection, MSCs were plated in enriched DMEM medium. The MSCs were then incubated for 24 hours with Tumor CM from MDA-MB-231 cells transfected by control vector or by TNF-α-expressing vector, or with enriched DMEM medium as control. Part of the above MDA-MB-231 Tumor CM was frozen in −20°C, as was also the case for CM of MSCs alone. All CM was then thawed at the same time (from MSCs stimulated with supernatants of MDA-MB-231 cells transfected by control vector or by TNF-α-expressing vector, from control MSCs and the CM of the tumor cells themselves). As appropriate, CCL2, CXCL8, CCL5 and TNF-α levels in these supernatants were determined by ELISA.

### Stimulation of patient CAFs by TNF-α and IL-1β

CAFs #1 and CAFs #2 isolates were cultured for 24 hours in CAF growth medium (as above). The cells were then washed twice in LPM starvation medium (Biological Industries), and incubated for 48 hours in LPM starvation medium in the presence of TNF-α (50 ng/ml), IL-1β (500 pg/ml) or cytokine vehicle (as above). CCL5 levels in the supernatants of the cells were determined by ELISA.

### Confocal analyses

Human BM-derived MSCs that were grown in the absence of or in the presence of MDA-MB-231-derived or MCF-7-derived Tumor CM for ~30 days (as above) were cultured for 24 hours on sterilized cover slips at 37°C. The cells were washed, fixed in 8% paraformaldehyde, permeabilized with 0.2% triton, blocked with 2% BSA in PBS, and then incubated with antibodies against αSMA (#A-2547; Sigma-Aldrich) and vimentin (#sc-6260; Santa Cruz Biotechnology, Santa Cruz, CA, USA). Negative controls included samples in which the primary antibodies were replaced by isotype-matched nonrelevant antibody controls (data not shown). The cells were then stained with the secondary antibodies, as appropriate. In parallel, cells were counterstained with the nuclear dye 4′,6-diamidino-2-phenylindole. Cell-coated cover slips were removed from the wells, embedded in mounting gel and imaged by confocal microscopy (Zeiss LSM 510; Carl Zeiss AG, Oberkochen, Germany).

### Polymerase chain reactions (PCR)

To determine the expression of human TNF receptors, total RNA was isolated by the EZ-RNA kit (#20-400-100; Biological Industries) from patient CAFs #1 and CAFs #2 isolates, from MSCs and from HL-60 cells that served as positive control. First-strand complementary DNA was generated using the M-MLV reverse transcriptase (#AM2044; Ambion, Austin, TX, USA). The expression of TNF-RI (TNFRSF1A) and TNF-RII (TNFRSF1B) was determined using the following primers: TNF-RI, forward 5′-GCACTGCCGCTGCCACACT-3′ and reverse 5′-AAGGCGATCTCGCAGGACG-3′, expected PCR product size 1,480 base pairs; and TNF-RII, forward 5′-ATGGCGCCCGTCGCCGTCT-3′ and reverse 5′-CCTGGTTAACTGGGCTTCATC-3′, expected PCR product size 1,390 base pairs. PCR amplification of TNF-RI was performed over 40 cycles (95°C for 20 seconds, 60°C for 20 seconds, 72°C for 90 seconds). PCR amplification of TNF-RII was performed over 40 cycles (95°C for 20 seconds, 65°C for 20 seconds, 72°C for 90 seconds). The sequence of the resulting PCR products was verified as TNF-RI and TNR-II. No-template controls were negative.

### Enzyme-linked immunosorbent (ELISA) assays

ELISA assays were performed in the linear range of absorbance using standard curves generated with recombinant proteins. The ELISA for CCL2 involved mouse monoclonal antibodies (mAbs) against human CCL2 (#500-M71; PeproTech) for coating, and biotinylated rabbit anti human CCL2 antibodies (#500-P34Bt; PeproTech) for detection. The ELISA for CXCL8 involved mouse mAbs against human CXCL8 (#511502; BioLegend) or rabbit polyclonal antibodies against human CXCL8 (#500-P28; PeproTech) for coating, and biotinylated goat anti human CXCL8 antibodies (#BAF208; R&D Systems) or biotinylated rabbit anti human CXCL8 antibodies (#500-P28Bt; PeproTech) for detection. The ELISA for CCL5 involved mouse mAbs against human CCL5 (#500-M75; PeproTech) for coating, and biotinylated goat anti human CCL5 antibodies (#BAF278; R&D Systems) for detection. The ELISA for TNF-α involved mouse mAbs against human TNF-α (#500-M26; PeproTech) for coating, and biotinylated rabbit anti human TNF-α antibodies (#500-P31ABt; PeproTech) for detection. Following the addition of streptavidin–horseradish peroxidase, the substrate TMB/E (Chemicon, Temecula, CA, USA) was added. The reaction was stopped by addition of 0.18 M H_2_SO_4_, and was measured at 450 nm.

### Immunohistochemistry

The expression of CCL2 by CAFs in biopsies of patients diagnosed with invasive ductal carcinoma (IDC) was determined as described previously [41]. Briefly, serial sections (5 μm thick) obtained from archived paraffin blocks of patients were processed and stained by antibodies against human CCL2. Staining was evaluated by a breast-cancer specialized pathologist.

### Western blotting

To determine the activation of activator protein 1 (AP-1) and NF-κB, TNF-α stimulation was carried out at time points that were determined by preliminary kinetics analyses (based on published literature and on our experience in other cell systems; data not shown). In all of the experiments described below, control cells were incubated with the relevant vehicles of the reagents. The following primary antibodies were used: phosphorylated p65 (#3033; Cell Signaling Technology, Danvers, MA, USA), total p65 (#4764; Cell Signaling Technology), phosphorylated c-Jun (#1527-s; Epitomics, Burlingame, CA, USA), total c-Jun (#610326; BD Transduction Laboratories, San Jose, CA, USA) and IκBα (#4814; Cell Signaling Technology). To determine the characteristics of patient CAFs and of Tumor CM-derived CAFs, the following primary antibodies were used: pan-cytokeratin (#MA5-13203; Thermo Fisher Scientific, Waltham, MA, USA), αSMA (#A-2547; Sigma-Aldrich and #ab5694; Abcam, Cambridge, UK), vimentin (#sc-6260; Santa Cruz Biotechnology), FSP1 (#ab12480; Abcam) and FAPα (#sc-135069; Santa Cruz Biotechnology). Loading controls were determined using antibodies against glyceraldehyde 3-phosphate dehydrogenase (GAPDH; #ab9485; Abcam), β-tubulin (#ab6046; Abcam) and Erk (#sc-154; Santa Cruz Biotechnology), as indicated in the figures. After washing, the membranes were incubated with the appropriate secondary antibodies. In all cases, conventional western blot procedures were taken and membranes were subjected to enhanced chemiluminescent (ECL) solution and X-ray film.

### Migration of monocytic cells

Monocytic THP-1 cells were grown in suspension in enriched RPMI medium and their migration in response to CM of MSCs, stimulated or not stimulated by TNF-α, was determined. To this end, MSCs were stimulated by TNF-α (50 ng/ml) for 24 hours, while control cells were exposed to the vehicle of the cytokine (0.1% BSA) in DMEM starvation medium. Supernatants from control and from stimulated cells were then collected and divided into groups as follows: supernatants from untreated control MSCs; supernatants from TNF-α-stimulated MSCs, treated by neutralizing antibodies against CCL2 (2 μg/ml, #MAB679; R&D Systems); and supernatants from TNF-α-stimulated MSCs, treated by isotype-matched nonrelevant antibody (2 μg/ml, #401201; BioLegend). Recombinant human CCL2 (rhCCL2) was used at 100 ng/ml as positive control in the migration assays. rhCCL2 was either untreated or treated by neutralizing antibodies against CCL2 (as above). All antibody treatments were performed for 30 minutes at 37°C. The migration of human THP-1 monocytic cells in response to these cell supernatants was assessed by a 48-well modified Boyden chamber through a polycarbonate polyvinylidene difluoride (PVDF) filter (8 μm pore size; Osmonics, Livermore, CA, USA). After 2 hours, the filters were fixed by methanol, and stained with a Diff-Quik kit (Dade Behring, Dudingen, Switzerland). The cells were counted in five high-power fields (HPF) by light microscopy in triplicate.

### Statistical analyses

**Figure 1:**
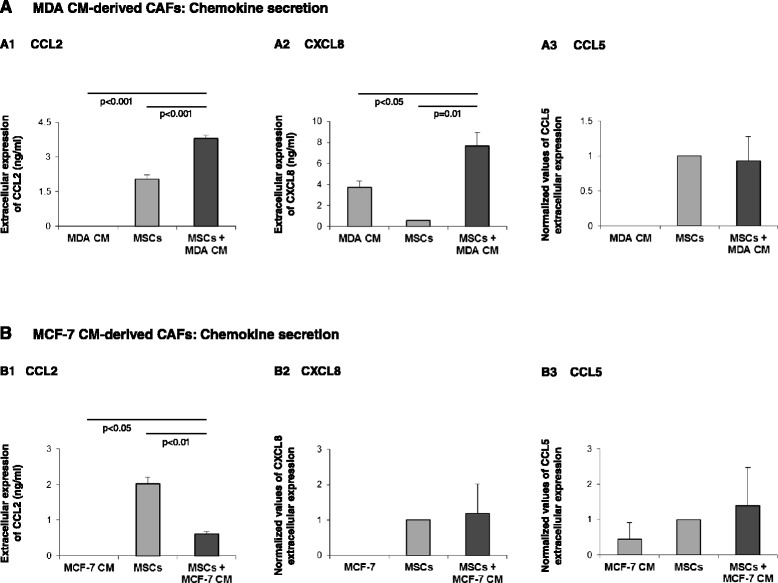
Impact of prolonged stimulation by Tumor CM on the release of inflammatory chemokines by the resulting CAFs. Human BM-derived MSCs were cultured with Tumor CM from MDA-MB-231 cells (MDA) **(A)** or MCF-7 cells **(B)** over a prolonged period of time (~30 days; MSCs + MDA CM or MSCs + MCF-7 CM, respectively). Twenty-four hours after medium exchange to fresh Tumor CM, cell supernatants were collected and the expression of CCL2 (A1, B1), CXCL8 (A2, B2) and CCL5 (A3, B3) was determined in comparison with supernatants of MSCs that were not supplemented with CM (MSCs) and with the original Tumor CM of MDA-MB-231 or MCF-7 cells alone (MDA CM or MCF-7 CM, respectively). Chemokine expression was determined by ELISA, in the linear range of absorbance. (A1), (A2), (B1) Representatives of *n* = 3 independent experiments that have shown similar results. (A3), (B2), (B3) Ratios between MSCs and MSCs + Tumor CM were not consistent in different experimental repeats. Therefore, in these panels, the findings are presented as mean ± standard deviation of normalized values (MSCs were given the value of 1) obtained in relevant experimental repeats (at least *n* = 3).

Student’s *t* test was used to calculate *P* values for ELISAs and chemotaxis assays. *P* <0.05 was considered significant. Adjustment for multiplicity of comparisons was done using the Benjamini–Hochberg procedure. Using this procedure, all of the significant results that were presented in the manuscript remained statistically significant after correcting for their multiplicity. The ELISA results presented in the paper show a representative experiment out of *n* ≥ 3 independent experiments that have shown similar trends. An exception is shown in Figure 1A3,B2,B3, where the effect of Tumor CM on chemokine release was not consistent; therefore, in these cases, the results are presented as mean ± standard deviation of the different experiments (MSCs were given the value of 1). The confocal pictures are representatives of many pictures that were taken in at least three independent experiments.

## Results and discussion

### The effects of long-term stimulation of MSCs by Tumor CM on the inflammatory nature of the resulting CAFs

Published reports indicate that prolonged stimulation of BM-derived human MSCs with breast tumor-derived factors (Tumor CM) has led to conversion of MSCs into functional CAFs that promoted tumor growth [11-14]. We began this study by determining the influence of such tumor-derived factors on the inflammatory traits of CAFs generated by MSCs exposed to Tumor CM, using the expression of the inflammatory/pro-malignancy chemokines CCL2, CXCL8 and CCL5 as readouts. To this end, we followed our published procedure on generation of CAFs that are functional *in vivo* in promoting breast cancer [13], by stimulating MSCs with Tumor CM for ~30 days. Following such stimulation with Tumor CM of MDA-MB-231 and MCF-7 breast tumor cells, the resulting cells have undergone conversion to CAF-like cells (to be termed herein CAFs), as expected (Figure S2 in Additional file 1) [12,13,82,83].

The generation of such CAFs by Tumor CM has been only partly accompanied by enhanced inflammatory profile of the resulting CAFs. Tumor CM of MDA-MB-231 cells has increased the release of CCL2 and CXCL8 but not of CCL5 (Figure 1A), while Tumor CM of MCF-7 cells did not promote the release of CXCL8 and CCL5 by the Tumor CM-generated CAFs (Figure 1B2,B3) and downregulated the expression of CCL2 by the cells (Figure 1B1). These results indicate that when CAFs are generated by exposure to breast tumor-derived factors, the process leads to only marginal increases in the inflammatory phenotype of the resulting CAFs. Moreover, only the CM of the more aggressive MDA-MB-231 breast tumor cells upregulated chemokine production by the resulting CAFs, revealing heterogeneity in the content of the factors produced by different breast tumor cells and in their impact on the expression of inflammatory traits by CAFs. The content of Tumor CM is complex (for example [6,62,84]); thus, the identity of the factors regulating the expression of the different chemokines is yet to be explored and the question of whether these same factors also induce the conversion of MSCs to CAFs needs to be addressed in future studies.

### TNF-α prominently induces the release of pro-cancerous chemokines by Tumor CM-generated CAFs and by patient CAFs

**Figure 2:**
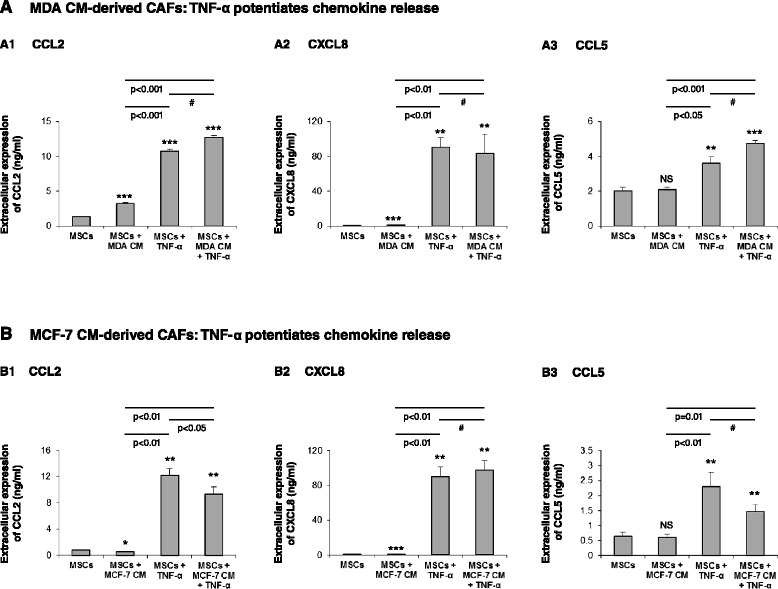
TNF-α induces potent elevation in inflammatory traits in MSCs and Tumor-CM-generated CAFs. Human BM-derived MSCs were cultured with Tumor CM from MDA-MB-231 cells (MDA) **(A)** or from MCF-7 cells **(B)** over a prolonged period of time (~30 days). TNF-α (50 ng/ml) or its vehicle (in control cells) was added for the last 24 hours. Expression of CCL2, CXCL8 and CCL5 was then determined in supernatants of the cells. (A) Expression of the chemokines in the four experimental groups included in the study: MSCs grown in culture for ~30 days without any additional stimulus (MSCs); MSCs grown in culture for ~30 days in the presence of Tumor CM derived from MDA-MB-231 cells (MSCs + MDA CM); MSCs grown in culture for ~30 days and stimulated by TNF-α at the last 24 hours of culture (MSCs + TNF-α); and MSCs grown in culture for ~30 days in the presence of Tumor CM derived from MDA-MB-231 cells and stimulated by TNF-α at the last 24 hours of culture (MSCs + MDA CM + TNF-α). Expression of CCL2 (A1), CXCL8 (A2) and CCL5 (A3) was determined by ELISA, in the linear range of absorbance. **(B)** Experimental design as in (A), but with MCF-7-derived CM. In each panel, the findings are representatives of at least *n* = 3 experiments that have shown similar results. In comparisons between MSCs and all other groups: *P <0.05, **P ≤0.01, ***P ≤0.001. NS, not significant. ^#^Differences between the two indicated groups have shown variability in *n* ≥ 3 independent experiments and overall were not significant.

TNF-α and IL-1β are highly expressed by cancer cells of patients in human breast tumors (and also by adjacent stromal cells and leukocytes). Of the two cytokines, TNF-α is of particular interest because of its divergent and at times opposing roles in the TME, and also because it induces the release of inflammatory chemokines by breast tumor cells and normal tissue cells (for example [41,85-87]). Also, as we show in Figure S3 in Additional file 1, the expression of TNF-RI and TNF-RII was denoted in MSCs. Thus, we questioned what is the impact of TNF-α on the inflammatory traits of CAFs derived from MSCs stimulated by Tumor CM. Breast tumor cells are known to lose in culture the capacity to release endogenous inflammatory cytokines, such as TNF-α or IL-1β (lack of endogenous TNF-α expression is shown in Figure S4 in Additional file 1, under nontransfected and control vector cells; results for IL-1β are not shown). Therefore, we have used exogenous TNF-α for stimulating the CAFs that were generated from MSCs by Tumor CM. We then determined the release of the inflammatory chemokines by the stimulated cells (Figure 2).

To determine the effects of TNF-α stimulation on Tumor CM-generated CAFs, MSCs that were exposed to Tumor CM for ~30 days were stimulated by TNF-α during the last 24 hours of culture (MSCs + MDA CM + TNF-α or MSCs + MCF-7 CM + TNF-α) followed by determination of CCL2, CXCL8 and CCL5 expression in cell supernatants. In parallel, chemokine expression was determined in MSCs that were exposed to Tumor CM alone for the same prolonged time period (MSCs + MDA CM or MSCs + MCF-7 CM) and in MSCs that were grown in culture for ~30 days without Tumor CM and were then stimulated by TNF-α during the last 24 hours (MSCs + TNF-α). The results shown in Figure 2 demonstrate that when CAFs were generated by prolonged exposure of MSCs to Tumor CM, the resulting CAFs responded to TNF-α by elevated release of CCL2, CXCL8 and CCL5. In parallel, stimulation of MSCs by TNF-α has also induced the release of the three chemokines (Figure 2). However, there were no significant differences in chemokine levels induced by stimulation of Tumor CM-generated CAFs with TNF-α when compared with stimulation of MSCs by TNF-α (two right-hand bars in Figure 2). These results indicate that Tumor CM did not cooperate with TNF-α in upregulating chemokine release and did not have much of an added value. TNF-α thus had a dominant role in inducing the inflammatory traits in Tumor CM-generated CAFs and in MSCs. Moreover, TNF-α induced the release of the inflammatory chemokines even when the Tumor CM alone (as shown in Figure 1 for CCL5 stimulation by MDA CM and for CXCL8 and CCL5 stimulation by MCF-7 CM) had no effect. These results indicate that the different chemokines are commonly regulated by TNFα, unlike their differential response to breast Tumor CM.

These findings suggest that, following their recruitment to the tumor site, MSCs are exposed to tumor constituents that promote their differentiation to CAFs and to some extent can also elevate their ability to release pro-malignancy chemokines. However, since the TME is enriched with TNF-α released by the tumor cells, this inflammatory cytokine turns into a most powerful inducer of chemokine release by CAFs. TNF-α thus enriches the TME with high levels of inflammatory/tumor-promoting chemokines presenting many deleterious pro-cancerous effects.

**Figure 3:**
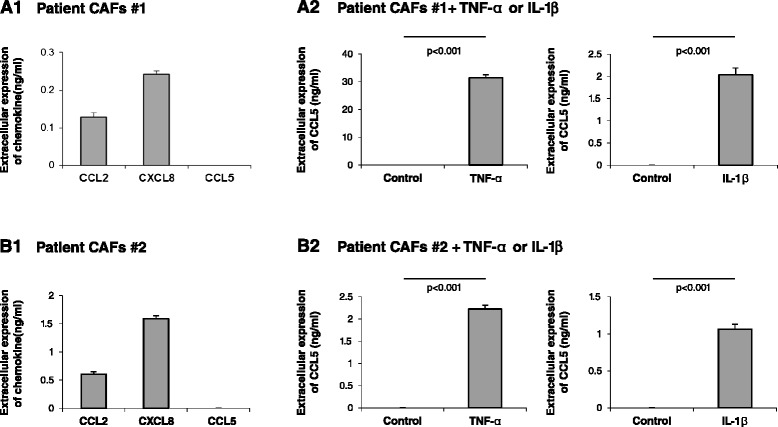
Basal and cytokine-induced release of inflammatory chemokines by patient CAFs. CAFs were isolated from lung metastasis of a breast cancer patient (CAFs #1) or from a primary tumor of a different breast cancer patient (CAFs #2). **(A1, B1)** Expression of the pro-malignancy chemokines CCL2, CXCL8 and CCL5 was determined by ELISA, in the linear range of absorbance. **(A2, B2)** Expression of CCL5 was determined following TNFa and IL-1β stimulation (TNF-α, 50 ng/ml; IL-1β, 500 pg/ml; 48 hours). Control cells were stimulated by vehicle. Expression of the chemokines was determined by ELISA, in the linear range of absorbance. In all panels, the findings are representatives of at least *n* = 3 independent experiments that have shown similar results.

To examine the potential clinical relevance of these observations we tested the content of inflammatory chemokines in CAFs obtained directly from a lung metastasis of a breast cancer patient (CAFs #1) and from a different patient’s primary breast tumor (CAFs #2). Two such CAF isolates showed production of CCL2 and of CXCL8 (Figure 3A1 and 3B1, respectively). Both patient CAF isolates did not secrete CCL5 (Figure 3A1,B1).

To better understand the regulation of chemokine release by patient CAFs, we asked whether CCL5 – which was not released constitutively by these cells – would be upregulated by inflammatory cytokines such as TNF-α and IL-1β that are known to prevail at the TME of breast tumors. Indeed, in line with the expression of both TNF-RI and TNF-RII by the two patient CAF isolates (Figure S3 in Additional file 1), both isolates responded to TNF-α with increased release of CCL5 (Figure 3A2,B2). Similar upregulation of CCL5 expression was observed after stimulation by IL-1β (Figure 3A2,B2), suggesting that chemokine induction by inflammatory cytokines may be a general effect and not necessarily limited to TNF-α. These findings support the clinical relevance of our previous findings, showing that TNF-α upregulated CCL5 in CAFs generated from MSCs by MDA Tumor CM (Figure 2A3) and by MCF-7 Tumor CM (Figure 2B3), and suggest that they reflect the clinical conditions of breast cancer.

Overall, the results obtained in this part of the study indicate that while tumor constituents had marginal effects on the inflammatory traits of Tumor CM-generated CAFs, the effects of the inflammatory microenvironment – represented here by TNF-α – were more prominent and may lead to pronounced upregulation of inflammatory chemokines in the TME. TNF-α and IL-1β highly prevail in breast tumors, and thus the inflammatory phenotype gained by patient CAFs in response to such inflammatory cytokines may form the basis of inflammation–stroma associations that promote breast cancer progression in patient tumors.

### TNF-α amplifies the inflammatory phenotype of MSCs by activating TNF-RI and TNF-RII

**Figure 4:**
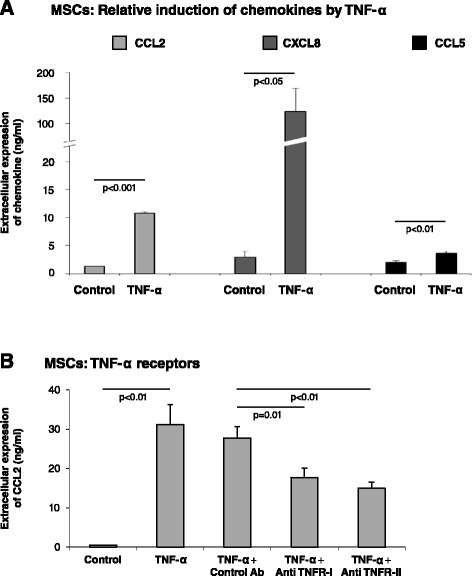
TNF-α upregulates the inflammatory profile of MSCs *via* receptors TNF-RI and TNF-RII. **(A)** Relative induction of CCL2, CXCL8 and CCL5 in MSCs by TNF-α. Human BM-derived MSCs were stimulated by TNF-α (50 ng/ml) for 24 hours and the expression of the inflammatory chemokines CCL2, CXCL8 and CCL5 in supernatants of MSCs was determined by ELISA, in the linear range of absorbance. Results are from an experiment in which all three chemokines were analyzed in parallel, and the ratios between the three chemokines are representatives of the values obtained in many experimental repeats. **(B)** Human BM-derived MSCs were exposed to neutralizing antibodies against tumor necrosis factor receptors TNF-RI and TNF-RII or nonrelevant isotype-matched control antibodies (Control Ab) 1 hour prior to stimulation with TNF-α (50 ng/ml), and in the course of 24 hours of stimulation by the cytokine. Control, cells stimulated with vehicle. Expression of CCL2 was determined by ELISA, in the linear range of absorbance. The findings are representatives of at least *n* = 3 independent experiments that have shown similar results.

MSCs that populate tumors are candidates for activation by inflammatory factors that are present at the TME. The findings shown in Figure 4A indicate that, following stimulation by TNF-α, CXCL8 was the chemokine most prominently induced in MSCs, followed by CCL2 and then CCL5, which underwent relatively low levels of induction. Moreover, the elevations in CCL2, CXCL8 and CCL5 production obtained in TNF-α-stimulated MSCs were recapitulated by CM of MDA-MB-231 breast tumor cells that were transfected to express TNF-α (Figure S5 in Additional file 1). Specifically, TNF-α expression by the tumor cells led to prominent upregulation of CCL2, CXCL8 and CCL5 production by MSCs (Figure S5 in Additional file 1). The inflammatory impact was not limited to TNF-α but was also detected following stimulation of MSCs by exogenous IL-1β (Figure S6 in Additional file 1). Overall, all three chemokines – CCL2, CXCL8 and CCL5 – were induced in MSCs by the inflammatory cytokines TNF-α and IL-1β, suggesting that they all may be upregulated in the TME by inflammatory mediators that are highly prevalent at breast tumor sites.

**Figure 5:**
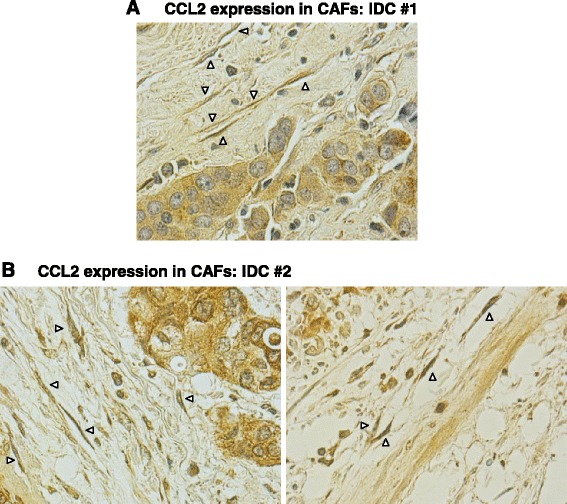
CCL2 is expressed by CAFs localized in vicinity to breast cancer cells in tumors of invasive ductal carcinoma (IDC) patients. CCL2 expression was determined by immunohistochemistry in biopsy sections of patients diagnosed with IDC: **(A)** IDC #1, **(B)** IDC #2. CCL2 staining was detected in cancer cells and in adjacent fibroblasts. Some of the CCL2-expressing CAFs are indicated by arrows.

Of the three chemokines induced in MSCs by TNF-α, CCL2 was of most interest to us. This chemokine is highly versatile and promotes the inflammatory and pro-cancerous nature of the TME by acting at many divergent levels, not all shared by CXCL8 and CCL5. Furthermore, CCL2 has very high relevance to our current study because we discovered that it was expressed by CAFs residing in proximity to human cancer cells in biopsies of invasive ductal carcinoma (IDC) patients (Figure 5). To further understand the molecular mechanisms mediating inflammation–stroma interactions, we focused on the mechanisms mediating the process of TNF-α-induced CCL2 secretion by MSCs. Using neutralizing antibodies to TNF-RI and to TNF-RII, the secretion of CCL2 was significantly inhibited compared with isotype-treated controls (Figure 4B; inhibition of TNF-RI gave rise to 36 to 55% reduction in CCL2 secretion and inhibition of TNF-RII to 34 to 54% reduction), indicating that both receptors mediate the effects of TNF-α on CCL2 release by MSCs (based on analyses combining the two antibodies at different concentrations (data not shown), we believe that the inhibition levels could not reach higher levels due to insufficient neutralizing activities of the antibodies). These findings are of major interest because TNF-RI is abundant in most cell types, while the expression of TNF-RII is not as promiscuous but nevertheless was found to be upregulated in breast cancer [44,88-91].

### Induction of inflammatory traits in MSCs by TNF-α is tightly regulated by the NF-κB pathway

**Figure 6:**
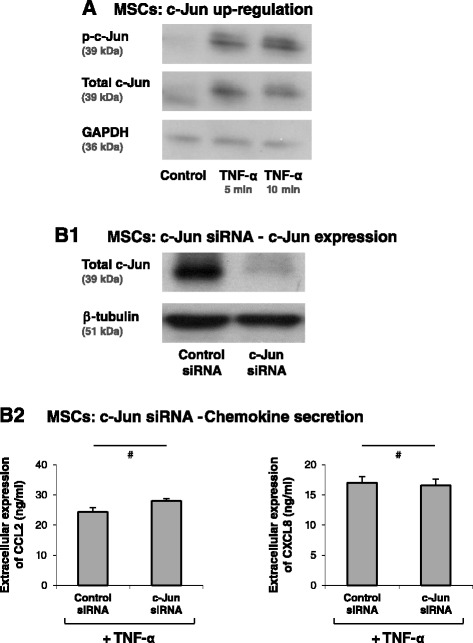
Induction of CCL2 and CLXL8 in TNFα-stimulated MSCs is not mediated *via* the AP-1 pathway. **(A)** Human BM-derived MSCs were stimulated by TNF-α (50 ng/ml) for 5 and 10 minutes. Control cells were treated by the vehicle of TNF-α. c-Jun levels and phosphorylation were determined by western blot (WB) analyses. Glyceraldehyde 3-phosphate dehydrogenase (GAPDH) was used as loading control. **(B)** Human BM-derived MSCs were transiently transfected by small interfering RNA (siRNA) to c-Jun or by control siRNA. (B1) c-Jun expression was determined by WB analyses. β-Tubulin was used as loading control. (B2) Following siRNA transfection, the cells were stimulated by TNF-α (25 ng/ml; in this part of the study we used a suboptimal concentration of TNF-α in order to facilitate detection of inhibitory effects) for 24 hours. Expression levels of CCL2 and CXCL8 in the supernatants of the cells were determined by ELISA, in the linear range of absorbance. ^#^siRNA to c-Jun has yielded minor increases or reductions in CCL2 and CXCL8 secretion in different experiments (see Results and discussion), and thus overall there was no significant effect on CCL2 and CXCL8 secretion. In all panels, the findings are representatives of *n* = 3 independent experiments that have shown similar results.

The AP-1 and NF-κB transcription factors are known to induce the expression of inflammatory chemokines in the immune context; however, their extent of involvement in inducing such chemokines depends on the cell type and stimulus. From the therapeutic perspective it is crucial to know whether both or only one of these transcription factors are mediating the response of MSCs to inflammatory stimuli that give rise to increased production of pro-malignancy chemokines. We began this part of the study by determining the involvement of AP-1 in induction of CCL2 and CXCL8, the two inflammatory chemokines that we found to be most relevant to the clinical setting, as shown by their expression by patient CAF isolates #1 and #2 (Figure 3). While AP-1 was activated by TNF-α stimulation of MSCs, it did not play a role in inducing CCL2 and CXCL8 (Figure 6). Specifically, in response to TNF-α, c-Jun – the active component of AP-1 – was rapidly induced in MSCs (possibly due to elevated stability), as was the phosphorylated form of c-Jun (Figure 6A). However, downregulation of c-Jun expression by siRNA (Figure 6B1) did not lead to reduction in CCL2 and CXCL8 expression following TNF-α stimulation (Figure 6B2; impact on CCL2 was in the range of 21% inhibition to 33% increase and for CXCL8 was in the range of 29% inhibition to 27% increase).

**Figure 7:**
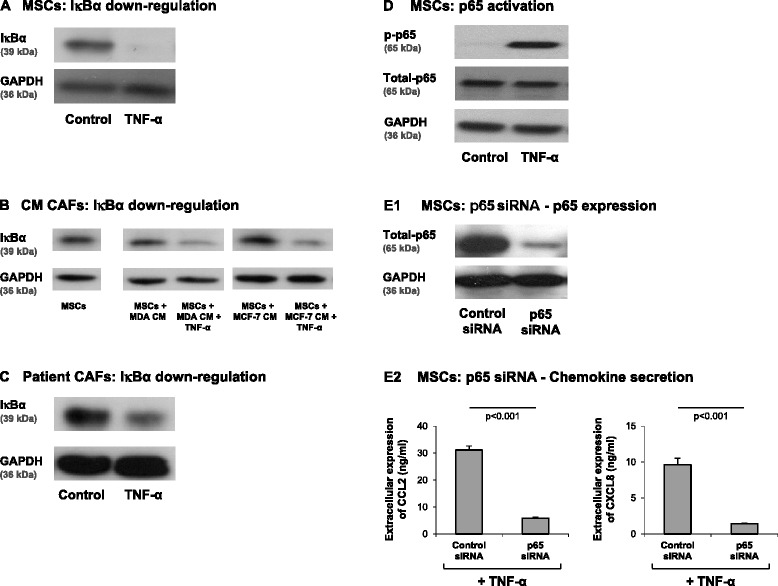
NF-κB is essential in mediating TNF-α-induced release of chemokines by MSCs. **(A)** Human BM-derived MSCs were stimulated by TNF-α (50 ng/ml) for 15 minutes. The levels of IκBα (the negative regulator of the NF-κB pathway) were determined by WB analyses. GAPDH was used as a loading control throughout. **(B)** CAFs were generated by culturing MSCs with Tumor CM from MDA-MB-231 (MDA) or MCF-7 breast tumor cells over a prolonged period of time (~30 days). TNF-α (50 ng/ml) was added for the last 24 hours to MSCs + Tumor CM cells and IκBα levels were determined by WB analyses. **(C)** CAF #1 cells were stimulated for 48 hours by TNF-α (50 ng/ml). IκBα levels were determined by WB analyses. **(D)** Human BM-derived MSCs were stimulated with TNF-α (50 ng/ml) for 10 minutes. p65 phosphorylation was determined by WB analyses. **(E)** Human BM-derived MSCs were transiently transfected by siRNA to p65 or by control siRNA. (E1) p65 expression was determined by WB analyses. (E2) Following siRNA transfection, the cells were stimulated by TNF-α (25 ng/ml; a suboptimal concentration of TNF-α in order to facilitate detection of inhibitory effects) for 48 hours. Expression of CCL2 and CXCL8 in the supernatants of the cells was determined by ELISA, in the linear range of absorbance. In all panels, the findings are representatives of *n* = 3 independent experiments that have shown similar results.

Different results were obtained for NF-κB, showing that this transcription factor is cardinal in CCL2 and CXCL8 upregulation by TNF-α in MSCs. First, we found that TNF-α stimulation induced significant reduction in the expression levels of IκBα, the negative regulator of the NF-κB pathway, in MSCs (Figure 7A). Similar results were obtained in TNF-α-stimulated Tumor CM-derived CAFs, as well as in patient CAFs (Figure 7B,C). In parallel, p65 was activated by TNF-α stimulation of MSCs (Figure 7D) and direct roles for NF-κB in CCL2 and CXCL8 induction were revealed when p65 was downregulated by siRNA to p65 (Figure 7E1). Specifically, p65 knockdown by siRNA resulted in almost complete inhibition of CCL2 (78 to 88%) and CXCL8 (82 to 90%) release by TNF-α-stimulated MSCs (Figure 7E2).

In contrast to the results obtained with TNF-α stimulation, a different pattern was obtained with regards to the impact of Tumor CM on the cells: while factors released by tumor cells resulted in MSC conversion to CAFs (Figure S2 in Additional file 1) and to elevated release of some of the inflammatory chemokines (Figure 1), no activation of the NF-κB pathway was observed in MSCs exposed to Tumor CM (Figure 7B). Here, we need to take into account the fact that the cells were exposed to Tumor CM for 1 month and thus it is possible that the rapid NF-κB activation signals could no longer be detected.

To summarize, the results of this part of the study clearly indicate an NF-κB-based pathway that regulates the expression of inflammatory and pro-malignancy chemokines such as CCL2 and CXCL8 in MSCs and in CAFs in response to TNF-α. NF-κB, but not AP-1, was involved in TNF-α-induced inflammatory patterns of CAFs and MSCs, despite the fact that the promoter domains of both these chemokines contain binding sites for c-Jun. This dichotomy between the NF-κB and AP-1 transcription factors in controlling CCL2 and CXCL8 production in MSCs indicates that the inflammatory profile of MSCs is tightly regulated. The preference of one transcription factor over another needs to be considered when potential therapeutic measures are directed to intracellular components involved in inflammation-induced tumor progression, such as NF-κB and AP-1.

### The inflammatory traits gained by MSCs following stimulation by TNF-α lead to potential pro-cancerous effects

Thus far, we have demonstrated that the inflammatory cytokines TNF-α and IL-1β induced inflammatory traits in CAFs and MSCs; furthermore, we have delineated the molecular mechanisms involved in these processes. To follow on these observations, we asked whether the inflammatory traits induced by TNF-α in MSCs have functional relevance to tumor-promoting events taking place at the TME.

**Figure 8:**
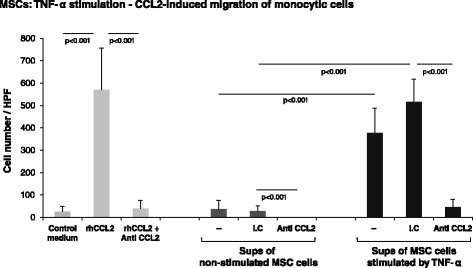
MSCs promote monocyte migration through TNF-α-induced secretion of CCL2. Supernatants (sups) were collected from human BM-derived MSCs that were stimulated by TNF-α (50 ng/ml) or by its vehicle for 24 hours. Thereafter, the sups were incubated with neutralizing antibodies for CCL2 or with nonrelevant isotype-matched control antibody (I.C.) for 30 minutes. The migration of monocytic cells in response to control medium (without chemokine), to recombinant human CCL2 (rhCCL2; 100 ng/ml) or to sups from TNF-α-stimulated or TNF-α nonstimulated MSCs was determined by Boyden chamber migration assays. Cells were counted under a high-power field (HPF). The findings are representatives of *n* = 3 independent experiments that have shown similar results.

CCL2 induces the recruitment to tumors of myeloid subpopulations that exert prominent pro-cancerous effects, including TAMs and MDSCs [17,67,71-75]. We examined the *in vitro* migration of monocytic cells and found that it was induced by supernatants of TNF-α-stimulated MSCs (Figure 8), which have been previously found to be enriched with CCL2 (Figures 2 and 4A). Furthermore, this migratory response was absolutely dependent on CCL2 because neutralization of this chemokine abolished the increase in monocytic cell migration in response to supernatants derived from TNF-α-stimulated MSCs (Figure 8). These results suggest that TNF-α, which is prevalent in the TME, acts on MSCs that reside in the tumors, leading to high production of CCL2 by these cells. This chemokine, in turn, may induce the recruitment of deleterious myeloid infiltrates to the tumor and thus further promote the malignant phenotype of the tumor. Chemokine pro-cancerous activities may thus not only be initiated by the tumor cells directly, but may also be induced by inflammatory factors of the TME acting on stromal cells residing in proximity to the cancer cells.

Furthermore, our findings suggesting that MSC-derived CCL2 promotes the recruitment of TAMs and MDSCs to breast tumors emphasize the important roles of inflammation–stroma interactions. These findings gain high relevance with the fact that CCL2 is indeed expressed in breast cancer cells, as we have shown in our study of biopsies of invasive ductal carcinoma patients (Figure 5).

## Conclusions

Much prominence has been given to inflammation-driven activities that promote tumor progression by acting on cancer cells, infiltrating leukocytes and adjacent stromal cells. In the current study we provide new findings on the networks that control the inflammatory phenotype of CAFs and MSCs, demonstrating key roles for the inflammatory cytokines TNF-α and IL-1β in upregulating the release of inflammatory, tumor-promoting chemokines by these two cell types.

TNF-α and IL-1β are highly relevant to the inflammatory setup of breast tumors [41-49]. They are minimally expressed by normal breast epithelial cells while their expression by breast tumor cells was detected in ~85% of breast tumors in patients; elevated incidence of TNF-α and IL-1β expression was highly correlated with relapsed and advanced disease [41-49]. In the current study we found that TNF-α upregulated the inflammatory phenotype of Tumor CM-derived CAFs and patient-derived CAFs, manifested by the increased release of CCL2, CXCL8 and CCL5 which are inflammatory chemokines having strong tumor-promoting activities in general and in breast cancer, particularly when derived from stroma cells [4,21,54-70]. TNF-α was also demonstrated to be a key inducer of inflammatory characteristics in MSCs, with activities that were much more pronounced and general than those induced by tumor constituents (Tumor CM). As a result of TNF-α stimulation, the stromal cells acquired the ability to release high levels of CCL2, CXCL8 and CCL5. The increased release of these chemokines from CAFs and MSCs may lead to exacerbated inflammatory and pro-cancerous nature of the TME and, based on our results (Figure 8), may lead to increased levels of deleterious myeloid infiltrates in breast tumors. The findings of our study thus suggest that stromal cells located near the cancer cells have a major role in promoting the inflammatory nature of the TME. Overall, a coordinated inflammatory network may be established at the tumor site between the cancer cells and stromal cells, set up by inflammatory cytokines such as TNF-α and IL-1β.

In this study we have also revealed the important role of NF-κB, but not of AP-1, in mediating the activities of TNF-α on MSCs and CAFs. These findings support other reports [10,92,93], indicating that the NF-κB pathway is a key cellular component that controls inflammatory events at the TME. The ultimate need for NF-κB activation in this process provides novel insights into specificity in regulatory pathways and may thus have clinical therapeutic implications. This need shows that NF-κB may be a preferred target for inhibition and that its roles are not limited to the tumor cells themselves and to infiltrating leukocytes [94,95], but rather extend to stromal cells at the tumor milieu. Thus, our study strongly supports the positioning of NF-κB as a master regulator that may serve as a therapeutic target, whose inhibition may lead to significant downregulation of inflammatory, pro-cancerous events in the entire tumor.

Currently, the inhibition of NF-κB in cancer has not entered the stage of clinical trials, and may be hampered by the fact that this pathway regulates a number of essential cellular processes and immune activities. Such hurdles can be overcome by directing the NF-κB-inhibiting modalities to specific target cells, in which this transcription factor is the key inducer of undesired inflammatory, tumor-promoting effects. In this respect, the results of our study imply that MSCs and CAFs could serve as appropriate targets for NF-κB inhibition, through which downregulation of the inflammatory TME could be achieved.

## Additional file

Additional file 1:
**Presents the following additional data.** **Figure S1.** verifying the purity and mesenchymal phenotype of patient-derived CAF isolates. **Figure S2.** showing that human BM-derived MSCs, exposed for ~30 days to Tumor CM, undergo transition to CAFs. **Figure S3.** showing that TNF-RI and TNF-RII are expressed by human BM-derived MSCs and by patient-derived CAFs. **Figure S4.** showing that breast tumor cells transfected with TNF-α-expressing vector release high TNF-α levels. **Figure S5.** showing that TNF-α produced by breast tumor cells induces chemokine release by MSCs. **Figure S6.** showing that stimulation by IL-1β upregulates the release of the inflammatory chemokines CCL2, CXCL8 and CCL5 by MSCs.

## Abbreviations

αSMA: α-smooth muscle actin
BM: bone marrow
BSA: bovine serum albumin
CAF: cancer-associated fibroblast
CM: conditioned medium
DMEM: Dulbecco’s modified Eagle’s medium
ELISA: enzyme-linked immunosorbent assay
FAPα: fibroblast activation protein alpha
FBS: fetal bovine serum
FSP1: fibroblast specific protein 1
GAPDH: glyceraldehyde 3-phosphate dehydrogenase
HPF: high power field
IL: interleukin
IDC: invasive ductal carcinoma
mAb: monoclonal antibody
MDSC: myeloid-derived suppressor cell
MSC: mesenchymal stem/stromal cell
NF: nuclear factor
siRNA: small interfering RNA
TAM: tumor-associated macrophage
TME: tumor microenvironment
TNF-α: tumor necrosis factor alpha
TNF-R: tumor necrosis factor receptor
WB: western blot
