# Supplementary material for: Regulation of the inflammatory profile of stromal cells in human breast cancer: prominent roles for TNF-α and the NF-κB pathway
Source: Stem Cell Res Ther. 2015 May 1;6(1):87. doi: 10.1186/s13287-015-0080-7 (PMC4469428; doi:10.1186/s13287-015-0080-7)
Supplement: Additional file 1: — Presents the following additional data. Figure S1. verifying the purity and mesenchymal phenotype of patient-derived CAF isolates. Figure S2. showing that human BM-derived MSCs, exposed for ~30 days to Tumor CM, undergo transition to CAFs. Figure S3. showing that TNF-RI and TNF-RII are expressed by human BM-derived MSCs and by patient-derived CAFs. Figure S4. showing that breast tumor cells transfected with TNF-α-expressing vector release high TNF-α levels. Figure S5. showing that TNF-α produced by breast tumor cells induces chemokine release by MSCs. Figure S6. showing that stimulation by IL-1β upregulates the release of the inflammatory chemokines CCL2, CXCL8 and CCL5 by MSCs. [file 13287_2015_80_MOESM1_ESM.pdf]

Fig. S1

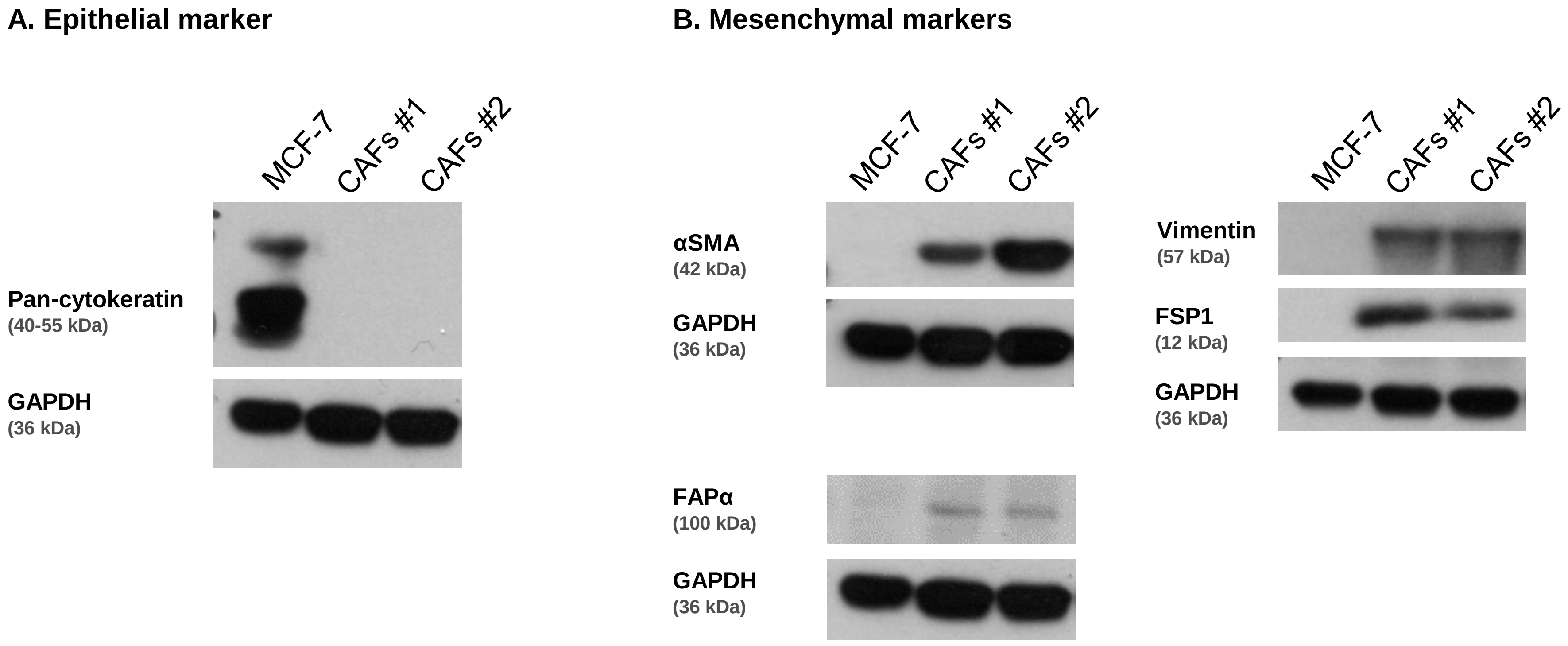

<u>**Supplementary Figure 1**</u>

Verifying the purity and mesenchymal phenotype of patient-derived CAF isolates

The purity of patient-derived CAF isolates was determined by WB analyses. **(A)** Epithelial marker: Cytokeratin. MCF-7 epithelial breast tumor cells served as positive control for cytokeratin expression (determined by pan-cytokeratin antibodies that detect proteins at diverse sizes). **(B)** Mesenchymal markers: αSMA, vimentin, FSP1, FAPα. MCF-7 cells served as negative control for the expression of mesenchymal markers. CAFs #1 cells were derived from a lung metastasis of a breast cancer patient and CAFs #2 cells were derived from a primary breast tumor of a different patient.

Fig. S2

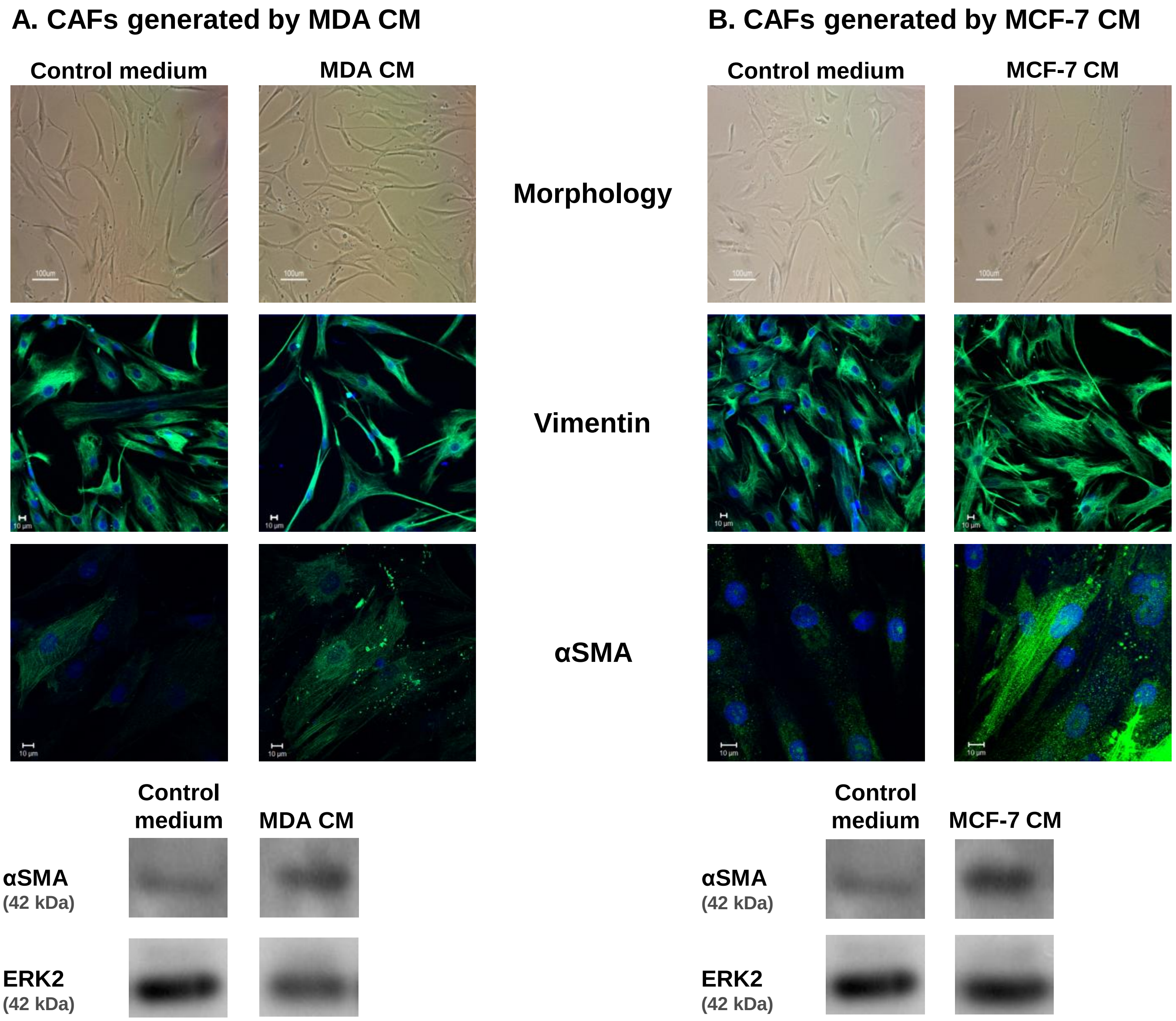

Supplementary Figure 2

Human BM-derived MSCs, exposed for ~30 days to Tumor CM, undergo transition to CAFs

Human BM-derived MSCs were stimulated by Tumor CM from **(A)** MDA-MB-231 cells (MDA) or from **(B)** MCF-7 cells for ~30 days in culture. Then, cell morphology was determined by light microscopy and in parallel, the expression of vimentin (shown at x25 magnification) and αMSA (shown at x63 magnification) was determined by confocal microscopy. Blue, DAPI staining. αSMA expression was also determined by WB analyses. The results are representatives of at least n=3 independent experiments that have shown similar results.

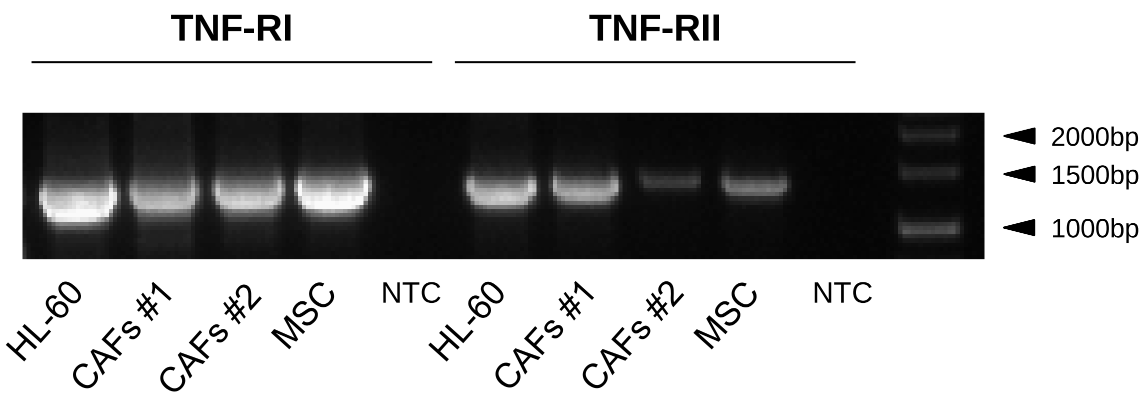

Supplementary Figure 3

TNF-RI and TNF-RII are expressed by human BM-derived MSCs and by patient-derived CAFs

Human BM-derived MSCs and patient-derived CAFs were analyzed for the expression of TNF-RI and TNF-RII by PCR. HL-60 cells served as positive control. The sequence of PCR-derived products was verified as TNF-RI and TNF-RII. NTC, No template control. CAFs #1 cells were derived from a lung metastasis of a breast cancer patient and CAFs #2 cells were derived from a primary breast tumor of a different patient.

Fig. S4

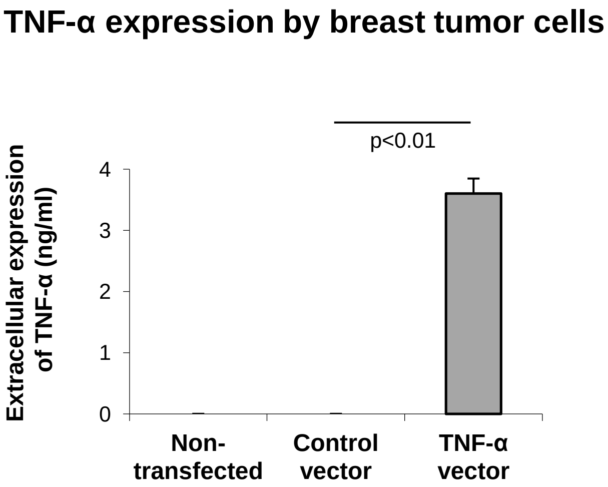

<u>Supplementary Figure 4</u>
Breast tumor cells transfected with TNF-α-expressing vector release high TNF-α levels

MDA-MB-231 breast tumor cells were transfected with vector expressing human TNF-α or with control vector. In parallel, the analysis included cells that were not transfected. The expression of TNF-α in CM of these cells was determined by ELISA, at the linear range of absorbance. The results are representatives of n=3 independent experiments that have shown similar results.

MSCs: Tumor-derived TNF-α - Chemokine secretion

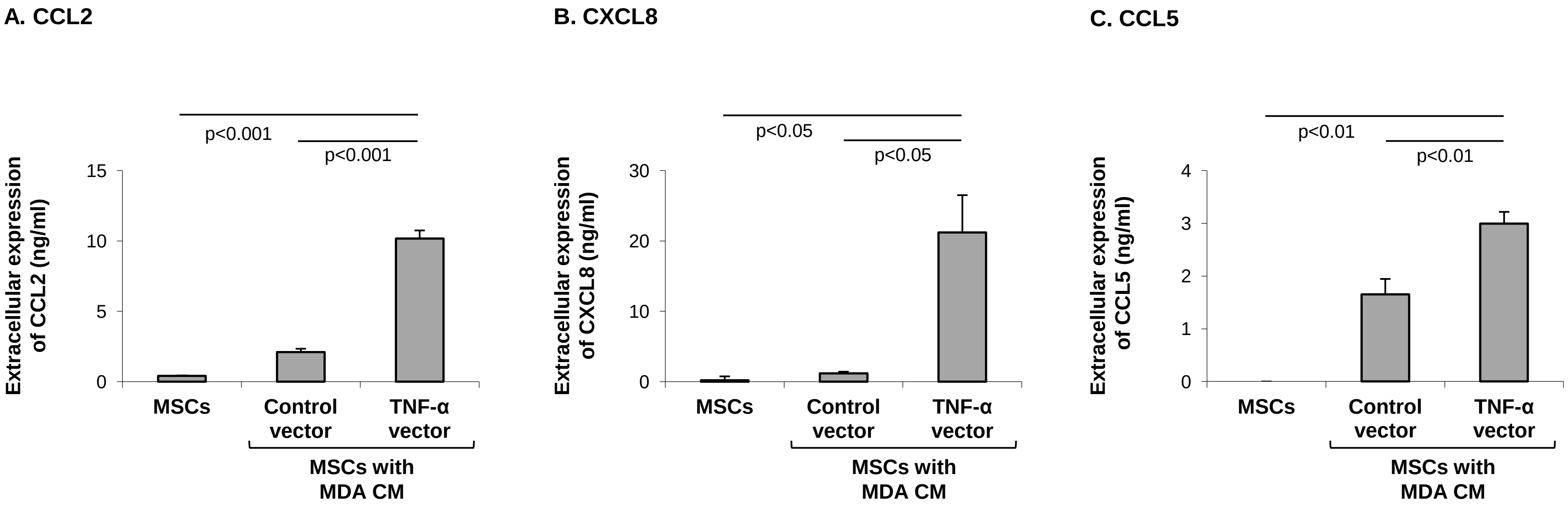

Supplementary Figure 5
TNF-α produced by breast tumor cells induces chemokine release by MSCs

Human BM-derived MSCs were cultured with CM from MDA-MB-231 (MDA) cells taken 24 hours following transfection of the MDA-MB-231 cells by control or TNF-α-expressing vector (TNF-α expression following transfection is shown in Supplementary Fig. S4). Control MSCs were cultured with regular growth medium. After 24 hours the expression of the pro-malignancy chemokines CCL2 **(A)**, CXCL8 **(B)** and CCL5 **(C)** was determined by ELISA, at the linear range of absorbance. The results are representatives of n=3 independent experiments that have shown similar results.

MSCs: IL-1β promotes chemokine secretion

A. CCL2 **B. CXCL8** **C. CCL5**

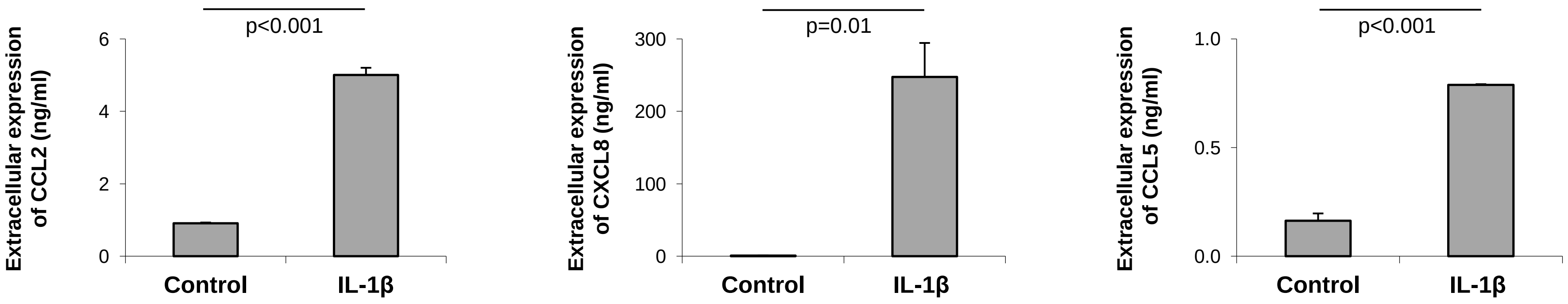

Supplementary Figure 6

Stimulation by IL-1β up-regulates the release of the inflammatory chemokines CCL2, CXCL8 and CCL5 by MSCs

Human BM-derived MSCs were stimulated by the cytokine IL-1β (500 pg/ml) for 24 hours. The expression of the inflammatory chemokines CCL2 **(A)**, CXCL8 **(B)** and CCL5 **(C)** in supernatants of MSCs was determined by ELISA, at the linear range of absorbance. In controls, we verified that the vehicle of the cytokine did not induce chemokine release by MSCs. The results are representatives of n=3 independent experiments that have shown similar results.
